# Stool and sputum microbiome during quinolone prophylaxis of spontaneous bacterial peritonitis: an exploratory study

**DOI:** 10.1186/s13099-020-00389-y

**Published:** 2020-10-30

**Authors:** Marcus M. Mücke, Sabrina Rüschenbaum, Amelie Mayer, Victoria T. Mücke, Katharina M. Schwarzkopf, Stefan Zeuzem, Jan Kehrmann, René Scholtysik, Christian M. Lange

**Affiliations:** 1 Present Address: Department of Internal Medicine 1, University Hospital Frankfurt, Goethe University, Theodor-Stern-Kai 7, 60590 Frankfurt am Main, Germany; 2 Present Address: Department for Gastroenterology and Hepatology, University Hospital Essen and University of Duisburg-Essen, Essen, Germany; 3 Institute of Medical Microbiology, University Hospital Essen, University of Duisburg-Essen, Essen, Germany; 4 Institute of Cell Biology, University Hospital Essen and University of Duisburg-Essen, Essen, Germany

**Keywords:** Quinolones, Multidrug-resistance, Infections, Bacterial abundance, Enterobacteriaceae

## Abstract

**Introduction:**

Quinolone prophylaxis is recommended for patients with advanced cirrhosis at high risk of spontaneous bacterial peritonitis (SBP) or with prior SBP. Yet, the impact of long-term antibiotic prophylaxis on the microbiome of these patients is poorly characterized.

**Methods:**

Patients with liver cirrhosis receiving long-term quinolone prophylaxis to prevent SBP were prospectively included and sputum and stool samples were obtained at baseline, 1, 4 and 12 weeks thereafter. Both bacterial DNA and RNA were assessed with 16S rRNA sequencing. Relative abundance, alpha and beta diversity were calculated and correlated with clinical outcome.

**Results:**

Overall, 35 stool and 19 sputum samples were obtained from 11 patients. Two patients died (day 9 and 12) all others were followed for 180 days. Reduction of Shannon diversity and bacterial richness was insignificant after initiation of quinolone prophylaxis (p > 0.05). Gut microbiota were significantly different between patients (p < 0.001) but non-significantly altered between the different time points before and after initiation of antibiotic prophylaxis (p > 0.05). A high relative abundance of *Enterobacteriaceae* > 20% during quinolone prophylaxis was found in three patients. Specific clinical scenarios (development of secondary infections during antibiotic prophylaxis or the detection of multidrug-resistant *Enterobacteriaceae*) characterized these patients. Sputum microbiota were not significantly altered in individuals during prophylaxis.

**Conclusion:**

The present exploratory study with small sample size showed that inter-individual differences in diversity of gut microbiota were high at baseline, yet quinolone prophylaxis had only a moderate impact. High relative abundances of *Enterobacteriaceae* during follow-up might indicate failure of or non-adherence to quinolone prophylaxis. However, our results may not be clinically significant given the limitations of the study and therefore future studies are needed to further investigate this phenomenon.

## Introduction

Infections in cirrhosis often lead to acute decompensation (AD) or acute-on-chronic liver failure (ACLF) and have been associated with increased morbidity and mortality [1, 2]. The most common infections in cirrhosis are spontaneous bacterial peritonitis (SBP), urinary tract infections and pneumonia [2, 3]. The bacteria causing SBP and numerous other systemic infections are believed to be enteric and to translocate through the intestinal barrier [4]. Therefore, antibiotic strategies to reduce the intestinal burden of pathogenic bacteria have been implemented and proven effective to prevent SBP and are currently recommended by national and international guidelines [5, 6].

Expanding this concept, Moreau and colleagues investigated the effectiveness of prophylactic quinolone therapy in patients with Child–Pugh C cirrhosis with and without ascites as to whether it might positively influence patients’ outcome. [7] Although the primary endpoint was not met (improvement of overall survival), they observed reduced infection rates, especially with regards to gram-negative bacteria, and an improved survival in a subgroup of patients with low ascites protein. Yet, the effectiveness of quinolone prophylaxis has been questioned as to whether all patients might benefit, especially those with known multidrug-resistant bacterial colonization or infection [8].

Several studies have linked the microbial composition of the gut and its degree of misbalance/dysbiosis to cirrhosis stage, its complication and patients overall survival [9, 10]. However, the role of the stool microbiome in cirrhotic patients is only partly understood. The use of antibiotics in these patients is believed to further influence bacterial composition. Yet, long-term studies on the effect and extent of these changes in patients with advanced liver disease on quinolone prophylaxis are lacking. The impact on orally administered prophylaxis on patients’ sputum microbiome has not been studied in cirrhotic patients so far. Moreover, altered bacterial functionality (e.g. endotoxemia) has been proposed to be an important co-factor. Some believe that stool RNA composition analysis might be an interesting alternative tool to further investigate and help discriminate between dead or viable bacteria in cirrhosis [11].

Aim of our explorative study was to investigate the impact of long-term quinolone antibiotic administration on microbial composition (i.e. decrease of bacterial richness or shift of relative abundance) of stool and sputum DNA and RNA as well as identify possible links to prophylaxis failure (i.e. infection) or MDRO development in patients with advanced stages of decompensated liver cirrhosis.

## Patients and methods

### Study design

Adult patients with liver cirrhosis, ascites and established indication for antibiotic prophylaxis to prevent SBP—according to the current German and the recently published European guidelines [5, 6]—were screened for this study between February 2017 and April 2018 in the Department of Internal Medicine I of the University Hospital Frankfurt, Germany. Patients were eligible for inclusion if they were not on antibiotic therapy and had not received antimicrobial agents in the last 2 weeks prior to study inclusion. Patients were excluded if they were younger than 18 years old, pregnant, diagnosed with hepatocellular carcinoma (HCC) beyond the Milan criteria, diagnosed with any malignancy other than HCC, treated with immunosuppressive agents, infected with human immunodeficiency virus, in the case of hypersensitivity or intolerance to quinolones, or if a previous episode of SBP with a quinolone resistant gram-negative rod was documented. Patients were excluded if they reported diarrhea within the last 5 days.

The diagnosis of liver cirrhosis was based on histology or by the combination of clinical, imaging and laboratory findings. SBP was defined as a neutrophil count in ascitic fluid of > 250/mm [3].

Primary or secondary antibiotic prophylaxis with quinolones was started at baseline in all patients at the time of study inclusion, either with norfloxacin 400 mg once daily or ciprofloxacin 500 mg once daily; the choice between both agents was made at the treating physicians’ discretion. Patients were part of the observational study on MDRO prevalence and occurrence of MDRO under SBP prophylaxis; details on the study protocol and clinical outcome are reported elsewhere [8]. In the present sub-analysis of this study, patients were included if they were willing to provide serial stool samples. Patients were followed for up to 6 months. Stool and sputum samples were obtained at baseline, 1 week, 4 weeks and 12 weeks thereafter. Patients were screened on the prevalence of MDRO colonization with nasal/oral and rectal swabs at baseline and during follow up. Patients were questioned on nutritional habits at baseline and during follow up visits with a standardized questionnaire (including number of meals, in-between snacks as well as the specific amount and type of meat, fish, eggs, dairy products, fruits vegetables, alcohol or sip feed nutritional products that were consumed). Patients were excluded if noticeable changes in nutritional habits were observed as judged by the treating physician.

Informed consent was obtained from participants and the ethical committee of the University of Frankfurt approved the study protocol (vote #452/16). The study was performed according to the Declaration of Helsinki.

### Sample preparation and analysis

Native stool and sputum samples were collected, immediately frozen and kept at − 80 °C. For DNA and RNA extractions, samples were thawed at 4 °C centrifuged for 10 min at 13,000×*g* and the supernatant was discarded. RNA was extracted with RNeasy PowerMicrobiome Kit (Qiagen, Venlo Netherlands) according to the manufacturer’s instructions. cDNA was created using GoScript™ Reverse Transcriptase Kit (Promega, Fitchburg, WI, USA) with a specific 1492R primer (5′-GGTTACCTTGTTACGACTT-3′) to amplify exclusively bacterial nucleic acids. Control PCRs for RNA samples were performed to detect any DNA contamination and RNA samples were treated with DNase treatment if DNA contamination was detected. DNA was extracted with QIAamp DNA Stool Mini Kit (Qiagen) according to the manufacturer’s instructions. Concentrations of nucleic acids were determined on a NanoDrop 2000 spectrophotometer (Thermo Fisher Scientific, Waltham, MA, USA).

In depth analysis of DNA and cDNA samples was performed by the Center for Metagenomics (CEMET GmbH, Tübingen, Germany): 16s RNA sequencing of bacterial DNA and cDNA was performed using the Illumina MiSeq platform (Illumina, San Diego, California, USA) with the MiSeq Reagent Kit v3 (600cycles) according to established protocols and the providers methods guide protocol [12, 13]. PCR amplification were performed with the following primers: Forward: 5′-CCTACGGGNGGCWGCAG-3′ and Reverse: 5′-GACTACHVGGGTATCTAATCC-3′ which target the hypervariable V3 and V4 region of the bacterial16S rRNA gene [14]. The FastQC control tool for high throughput sequence data was used for quality control [15]. Data merging and read trimming was performed with USEARCH and data was compared with the NCBI Bacterial 16s rRNA Database (for taxonomic classifications MALT) [16, 17].

### Statistics

We used demultiplexed paired-end fastq files and mapping files including metadata of the samples included in the study as input files for data analyses using QIIME2 (Quantitative Insights Into Microbial Ecology, Version 2019.4), a free, open-source and community developed next-generation microbiome bioinformatics platform [18]. Adapters and primers were removed using Cutadapt [19]. The DADA2 software package [20], included in QIIME2, was used for modeling and correcting Illumina fastq files including elimination of chimeras with the consensus method. We truncated 20 bases of the forward and 80 bases of the reverse reads because of the decreasing quality scores of bases at the end of the reads.

We used the q2-diversity plugin for computing different alpha diversity metrics, Shannon’s diversity index and observed OTUs, and beta diversity metrics using weighted and unweighted UniFrac distance matrices with a sampling depth of the sample with the lowest number of reads for each data set (4929 for extracted DNA from stool samples, 3995 for RNA extracted from stool samples and 2,679 for sputum samples). A Naïve Bayes classifier, trained on the Greengenes 13_8_99% OTUs 16S rRNA gene full length sequences with the q2-feature-classifier plugin, was used for assignment of taxonomy. We used ANCOM [21] for differential abundance testing. Associations between the categorical metadata columns and alpha diversity data were studied using the qiime diversity alpha-group-significance command. We used the qiime diversity beta-group significance command to perform permutational multivariate analysis of variance (PERMANOVA) of unweighted and weighted UniFrac distance matrices with 999 permutations to calculate p-values and test for significant differences in beta diversity among the groups.

Taxonomy, the biom-table generated from the QIIME2 DADA2 analyses, the Mapping file and weighted and unweighted distance matrices generated by QIIME2 were imported to Calypso software for further analysis and drawing of the figures [22]. Normalization of the data in Calypso was performed by total sum normalization (TSS) and without removing rare taxa.

## Results

### Patient characteristics

A total of 16 patients were eligible for inclusion in this study. Among these, one refused to take antibiotic prophylaxis, two were lost to follow-up, one withdrew consent and one developed an infection and required antibiotic therapy before inclusion. Overall, we obtained 35 stool samples and 19 sputum samples over the study period from 11 patients that could finally be analyzed.

Baseline characteristics of these patients are depicted in Table 1. Seven patients were male (63.6%). The majority suffered from alcoholic cirrhosis (81.8%). Of note, most patients had advanced liver cirrhosis as reflected by a high mean MELD score (20 ± 9) and a proportion of 72.7% of patients with Child–Pugh C cirrhosis. Interestingly, five patients had the indication for secondary SBP prophylaxis due to a prior SBP, yet had not received it before study inclusion. The majority of patients (72.7%) received norfloxacin to prevent SBP during the study period, while the remaining patients received ciprofloxacin. In the current study all but two patients (who died at day nine and twelve during follow-up) received antibiotic prophylaxis and were followed for the total of 180 days.

### Analysis of stool microbiota

As earlier proposed by some studies that stool RNA composition analysis might be an interesting alternative tool to further characterize the patients microbiome [11], we applied two approaches, analyzing both DNA and RNA content in the collected stool samples.

In the DNA-based microbiota analysis of 35 stool samples, we obtained 422,138 quality filtered reads with a mean sequence frequency of 12,061 reads, the maximum was 26,876 and the minimum number of sequences 4929; overall, we identified 2000 different isolates.

**Fig. 1:**
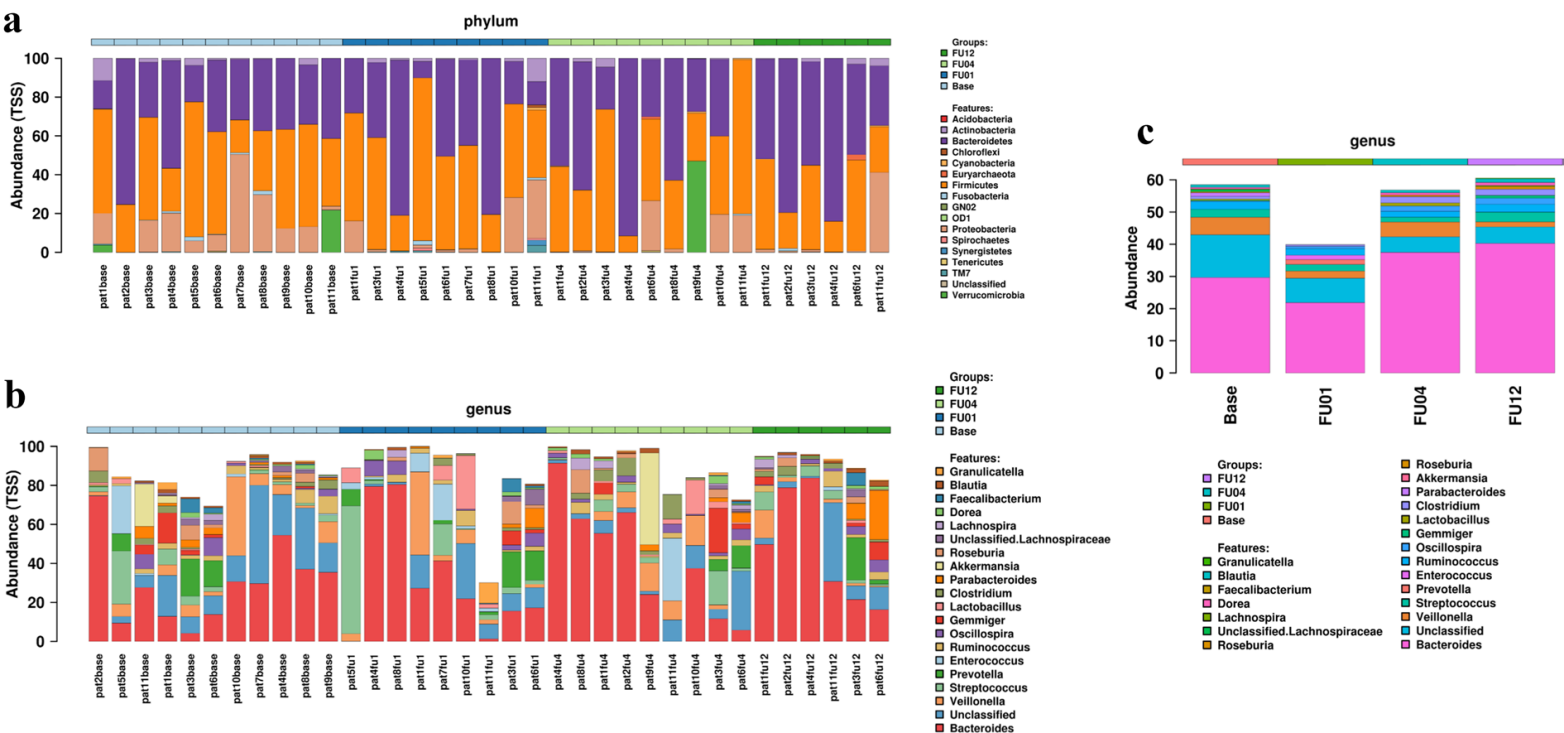
Inter- and intra-individual bacterial composition for stool DNA. Bacterial composition at baseline and during follow up is depicted at phylum level (**a**) and genus level (**b**). **c** Shows overall bacterial composition in all patients according to different time points at genus level. Note, at genus level, relative abundance of the 20 most abundant genera is presented. No significant differences were observed at genus level

Inter- and intra-individual bacterial compositions for stool DNA at baseline and during follow up are depicted in Fig. 1, at phylum level (Fig. 1a) and genus level (Fig. 1b). Figure 1c shows overall bacterial composition in all patients according to different time points at genus level. No significant difference of relative abundances was observed at genus level.

**Fig. 2:**
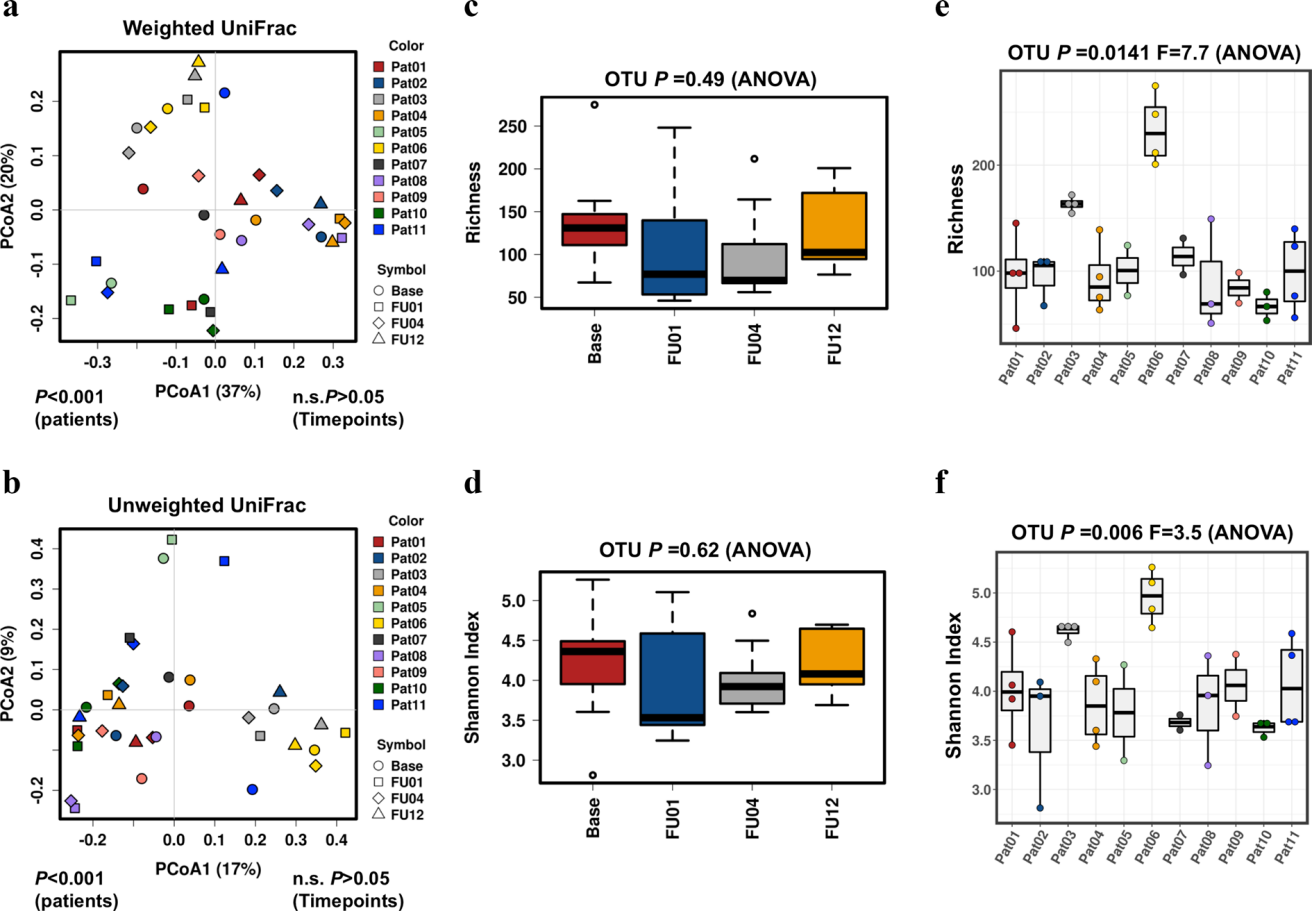
Principal coordinates analysis (PCoA) of weighted and unweighted UniFrac distances (**a**, **b**) as well as bacterial richness and Shannon’s index diversity with respect to different time points (**c**, **d**) and inter-individual differences (**e**, **f**) of stool DNA

When assessing alpha diversity (Shannon’s diversity index and bacterial richness) and beta diversity (weighted and unweighted UniFrac), gut microbiota were significantly different between patients (p < 0.001) but non-significantly altered between the different time points before and after initiation of antibiotic prophylaxis (p > 0.05, Fig. 2). We observed a reduction of Shannon diversity and bacterial richness after 1 and 4 weeks of prophylaxis, which recovered until week 12. However, changes were insignificant (p > 0.05). Similar results were obtained when stool RNA was analyzed (Additional file 1: Fig. S1).

### Analysis of *Enterobacteriaceae*

**Fig. 3:**
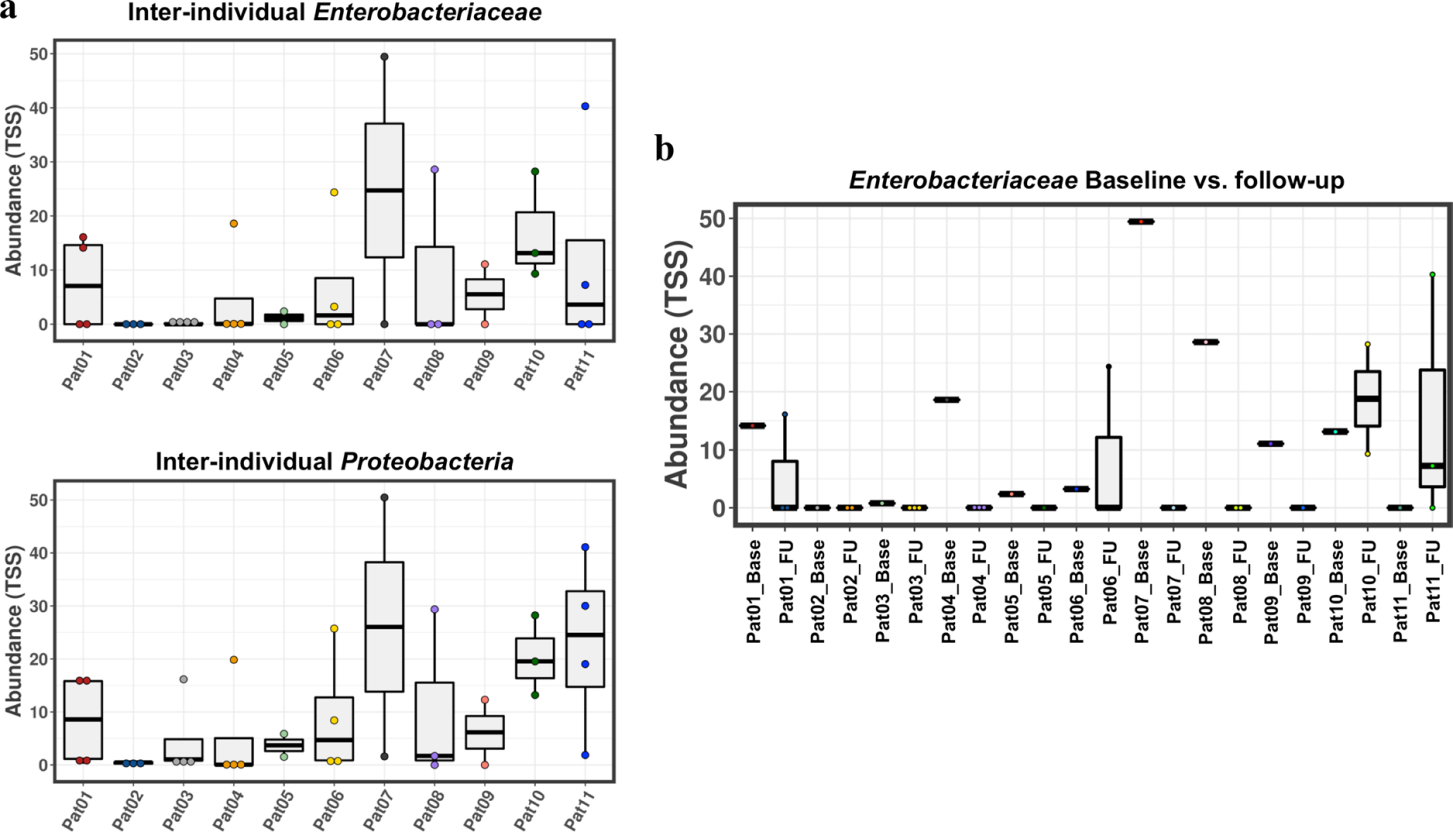
Relative abundance of *Enterobacteriaceae* and Proteobacteria in stool DNA (**a**) and changes of relative abundance of *Enterobacteriaceae* from baseline and during follow up (**b**). In patients that experienced a specific clinical scenario a high relative abundance of Enterobacteriaceae > 20% was observed during quinolone prophylaxis

Collectively, the above-described data showed a moderate impact of antibiotic prophylaxis on the global microbiome of patients. Since we observed an unusual high abundance of Proteobacteria in several patients and since the rationale of quinolone prophylaxis is targeting pathogenic gram-negative intestinal bacteria (in particular *Enterobacteriaceae*, phylum Proteobacteria) to prevent SBP, we analyzed the impact of quinolone prophylaxis on *Enterobacteriaceae* in more detail. Figure 3a shows relevant differences in the abundance of *Enterobacteriaceae* and at phylum level (Proteobacteria) between patients. In general, relatively high frequencies of *Enterobacteriaceae* were observed at baseline, which declined during antibiotic prophylaxis to undetectable levels in 8 out of 11 patients until week 12 of prophylaxis (Fig. 3b). However, in three patients an “incomplete response” with respect to the decline of *Enterobacteriaceae* was observed. In patient #6, *Enterobacteriaceae* decreased from 3.2 to 0% from baseline to FU week 1, and then increased to 24% at FU week 4 to decrease to 0% at FU week 12. In patient #10, *Enterobacteriaceae* comprised 13% of the stool microbiome at baseline and persisted at 9% in the last follow up sample. In patient #11, no *Enterobacteriaceae* were observed at baseline and FU week 1, but at FU week 4 we detected 7% *Enterobacteriaceae*, which further increased to 40% of the stool microbiome at FU week 12. Concordant changes were observed for the phylum Proteobacteria in these patients.

Importantly, these three patients were the only ones who had a high relative abundance of Enterobacteriaceae > 20% at one time-point during quinolone prophylaxis (Fig. 3b) and each of those time-points were associated with a specific clinical scenario: In patient #6, antibiotic prophylaxis was interrupted from FU week 3 until FU week 5 according to the suggestion of his general practitioner, he then developed a secondary infection (pneumonia with *Enterobacter cloacae*); in patient #10 quinolone-resistant *Enterobacteriaceae* were detected in baseline rectal swabs; and patient #11 developed a new quinolone- and carbapenemase resistant *Enterobacteriaceae* which was detected in FU4 and FU12 rectal swabs, he then developed a culture-negative sepsis.

### Analysis of sputum microbiota

In the DNA-based microbiota analysis of 19 sputum samples, we obtained 200,715 quality filtered reads with a mean frequency of 10,563, the maximum was 14,829 and minimum frequency 2679.

**Fig. 4:**
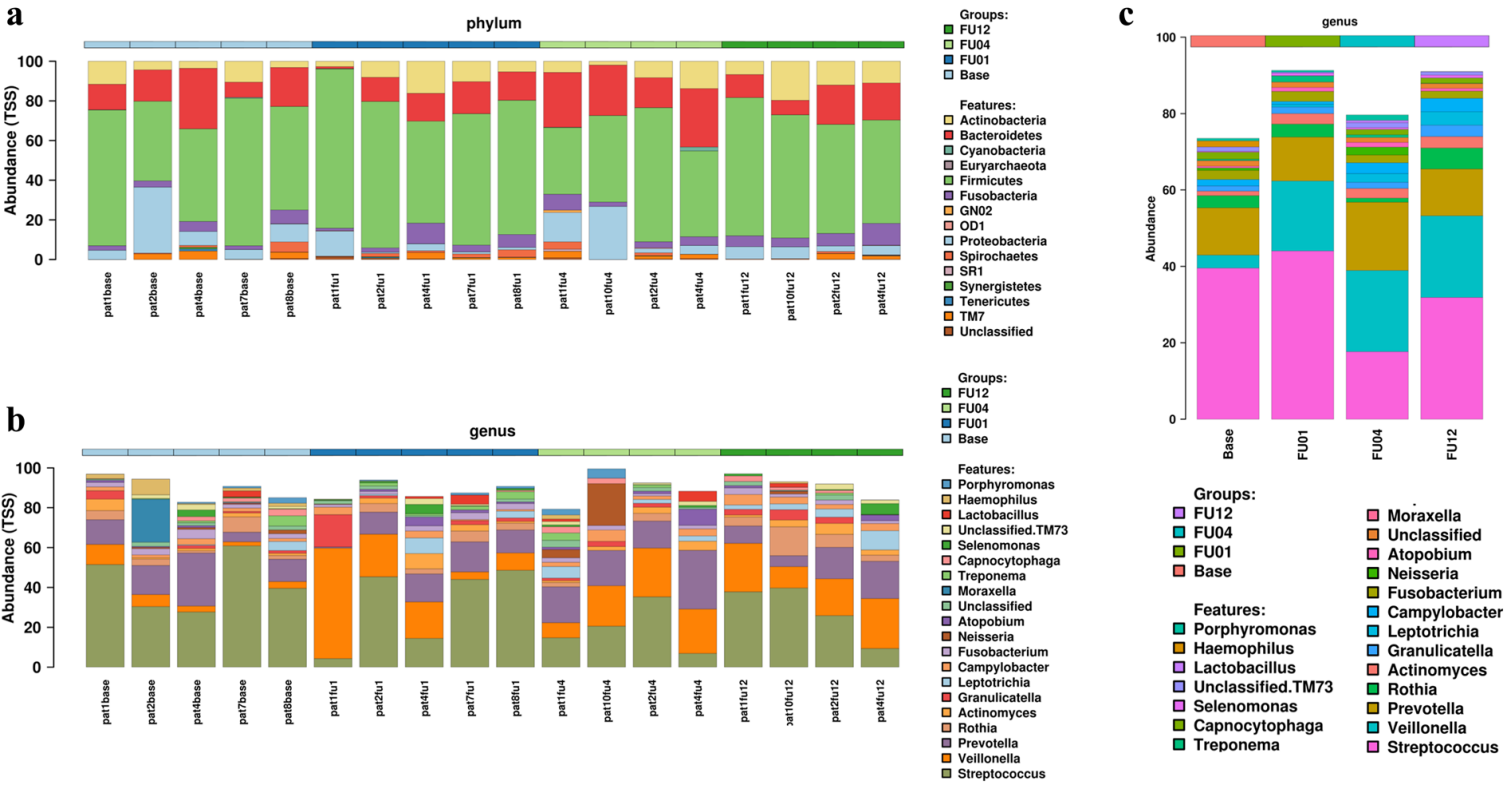
Inter- and intra-individual bacterial composition for sputum DNA. Bacterial composition at baseline and during follow up is depicted at phylum level (**a**) and genus level (**b**). **c** Shows overall bacterial composition in all patients according to different time points at genus level. Note, at genus level, relative abundance of the 20 most abundant genera is presented. No significant differences were observed at genus level

Inter- and intra-individual bacterial compositions for sputum DNA at baseline and during follow up are depicted in Fig. 4 at phylum level (Fig. 4a) and genus level (Fig. 4b). Firmicutes, Bacteroidetes and Actinobacteria were the most prevalent bacteria a phylum level, Streptococcus, Veilonella and Prevotella at genus level. Figure 4c shows overall bacterial composition in all patients according to different time points at genus level. No significant differences of relative abundances were observed at genus level.

**Fig. 5:**
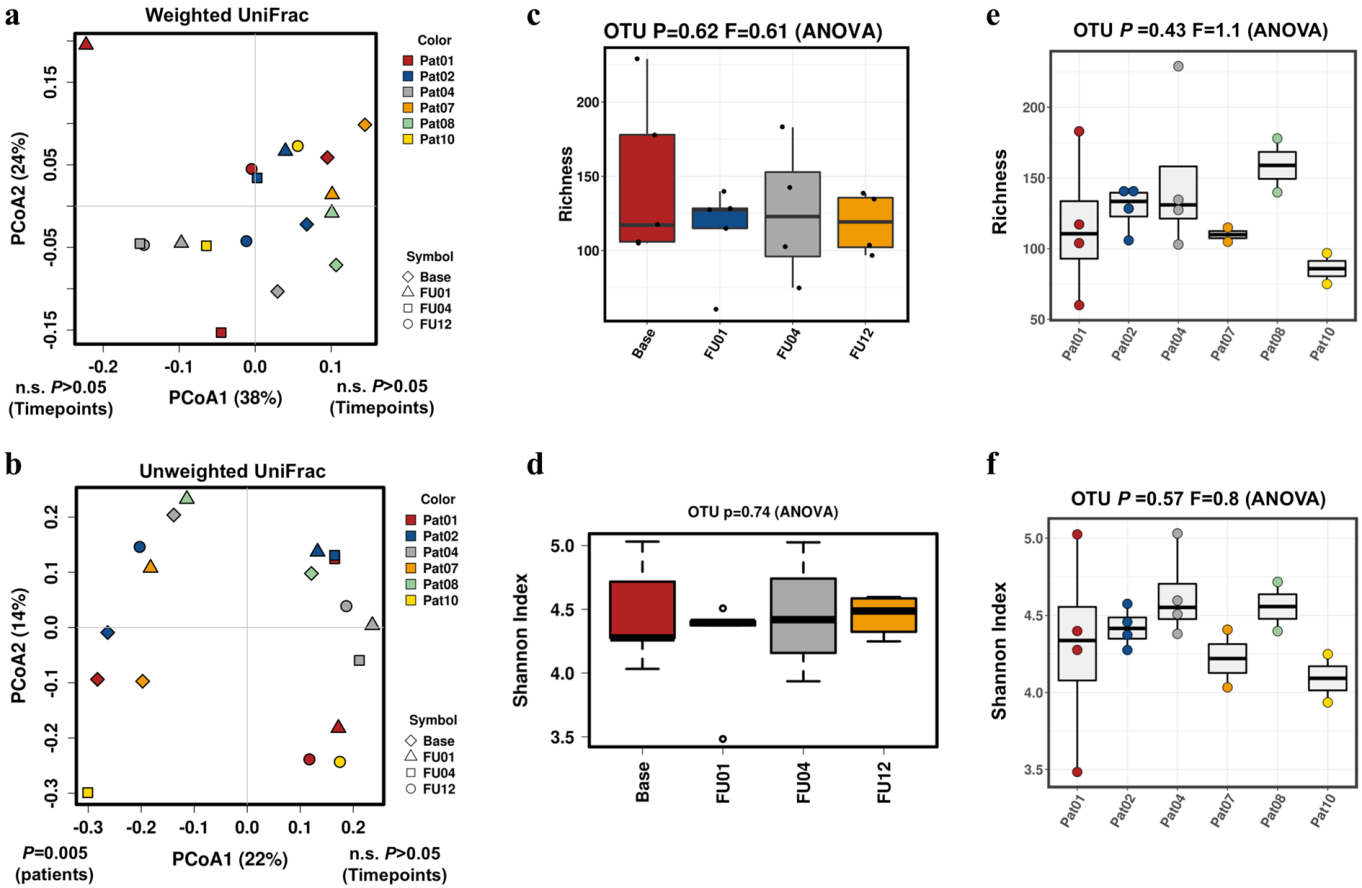
Principal coordinates analysis (PCoA) of weighted and unweighted UniFrac distances (**a**, **b**) as well as bacterial richness and Shannon’s index diversity with respect to different time points (**c**, **d**) and inter-individual differences (**e**, **f**) of sputum DNA

When assessing alpha and beta diversity sputum microbiota were comparable between patients and between the different time points before and after initiation of antibiotic prophylaxis (p > 0.05) with the exception of the unweighted UniFrac between individual patients (p = 0.005, Fig. 5). No change of Shannon diversity and bacterial richness after initiation of prophylaxis could be observed (p > 0.05).

## Discussion

In our study, we observed only a moderate impact of antibiotic prophylaxis on the gut and sputum microbiome in individual patients after the initiation of antibiotic prophylaxis. However, we found remarkable inter-individual differences of the stool microbiome between patients. Importantly, high frequencies of *Enterobacteriaceae* (> 20%) during antibiotic prophylaxis were associated with specific clinical scenarios such as the presence/occurrence of (multidrug-)resistant *Enterobacteriaceae* or the occurrence of infections.

Currently, there is an ongoing debate as to whether to extend the indication for antibiotic prophylaxis in cirrhosis: so far, it has been recommended for patients with ascites at high risk for SBP (primary prophylaxis) or with prior SBP (secondary prophylaxis) [5, 6]. We observed higher risk of prophylaxis failure in patients with known status of multidrug-resistant bacteria (MDRO) and a recent meta-analysis reported a reduced efficacy of quinolone prophylaxis to prevent SBP over the last decades [8, 23], while others have observed an alarming increase of MDRO in patients with cirrhosis over time [24, 25]. Yet, Moreau et al. [7] observed in a recently published, large randomized controlled trial significantly reduced overall infection rates in cirrhotic patients in general and a better overall survival in those with low ascites protein content. Taken together, on the one hand, we might see a future restriction of quinolone prophylaxis to prevent SBP in patients with MDRO but, on the other hand, a possibly broader indication for the prophylactic use in patients with cirrhosis in general. Thus, there is an unmet need to further understand the impact of long-term use of antibiotic administration in patients with liver cirrhosis, particularly with regards to changes of patients’ microbiome.

We observed rather minor intra-individual differences of the gut microbiome within individual patients at baseline and during antibiotic prophylaxis. The trend to lower alpha diversity metrics after 1 and 4 weeks after initiation observed in stool analysis was probably not significant due to the limited number of patients, yet alpha diversity recovered thereafter. So far, rifaximin, which is currently in use to prevent hepatic encephalopathy but can also be administered to prevent SBP, has been reported to have negligible effects on the patients’ microbiome [26, 27]. Yet, currently available data favors quinolones, especially norfloxacin, as the antibiotic of choice for SBP prophylaxis. Here we report that changes in the microbiome remained comparably small in patients on long-term quinolone prophylaxis. Similarly, in a recent globally conducted study on the emergence of quinolone and multidrug resistance, no independent association between the emergence of quinolone-resistance and the use of quinolone prophylaxis could be observed [25].

Similar to others [10], we found a high abundance of Proteobacteria at baseline prior antibiotic usage with especially high relative frequencies of *Enterobacteriaceae*. This is pivotal, as *Enterobacteriaceae* family includes *Escherichia coli*, *Klebsiella *spp. and other bacteria, which function as key pathogens causing infections in cirrhosis, above all SBP. During prophylaxis, relative abundance of Proteobacteria decreased, as well as frequencies of *Enterobacteriaceae*. Clinically, no infections were observed in these patients indicating a good effectiveness of quinolone prophylaxis.

In a patient who temporally discontinued prophylaxis as well as in two other patients with presence of MDRO an increase of Proteobacteria, especially *Enterobacteriaceae* (> 20%), during prophylaxis could be observed. Two of them developed infections. One could speculate that there are patients, possibly those who acquire or already are colonized with MDRO that might not benefit from quinolone prophylaxis in this setting. Vehreschild and others described this phenomenon as “colonization, domination, and infection” [28]. They postulated that the occasional acquisition of MDRO may lead to intestinal MDRO colonization and then, due to antibiotic exposure or other substances with an antibacterial effect, to intestinal domination of MDRO. Subsequently, these MDRO can possibly be harmful to their host and cause infections. In this scenario, a notably increase of *Enterobacteriaceae* (or Proteobacteria) during consecutive microbial stool analyses might be helpful in predicting prophylaxis failure. However, future studies are needed to confirm these findings and clarify if stool microbiome analysis can discriminate between those who benefit and those who do not benefit from prophylaxis.

Data of some studies suggest that qPCR of DNA may detect bacterial cells several days to weeks after a loss of viability after antibiotic treatment and therefore Bajaj and colleagues proposed to utilize bacterial RNA content analysis to better reflected their metabolic activity, as altered bacterial functionality (e.g. endotoxemia or the fecal bile acid profile) may have greater influence on patients’ outcomes rather than their composition [10, 29]. To date, confirmatory studies are pending. We therefore analyzed both stool DNA and RNA. In our analysis, described bacterial composition, their changes as well as alpha and beta diversities were comparable.

Moreover, analyses revealed only insignificant changes in bacterial sputum composition when assessing alpha and beta diversity with no changes of Shannon diversity and bacterial richness after initiation of prophylaxis. This is important to note, as pneumonia and respiratory infections are common among patients with liver cirrhosis, especially with ACLF [1, 2]. Thus, future studies may focus on gut/stool microbiome analysis when assessing the impact of long-term quinolone prophylaxis in these patients. Here, a special focus should also include the prevalence of resistance mechanisms in quinolone prophylaxis up to the complete resistome analyses using e.g. long read sequencing.

Our study has several limitations. The low number of patients with some of them missing follow ups does not allow us to draw universal conclusion and further studies are needed to confirm our results. Moreover, we had no control group in our study, as it was part of a prospective observational study, so no comparison, neither at baseline (e.g. healthy subjects or compensated cirrhotic patients) nor during follow up could be performed. However, this is the first pilot study in patients with advanced liver cirrhosis to report long-term effects of antibiotic prophylaxis and changes in bacterial composition that seem to be associated with prophylaxis failure. Additionally, we used both DNA and RNA microbiome analysis to overcome possible confounding with non-viable bacteria. Furthermore, due to the high rate of infections in patients with end-stage liver disease, we included patients who did not receive antibiotic therapy in the last 2 weeks prior study inclusion (and not rather 3–6 months), which might be too short to mitigate all effects of a possible prior antibiotic use. Similarly, the usage of proton-pump inhibitors (PPIs) could pose a possible bias [30]. However, a majority of patients, received PPIs in this trial (and has received them long before study inclusion). Patients continued to take PPIs throughout the study, thus, the effect, especially on changes of the microbiome during the observation period, might be less influential.

## Conclusion

Taken together, in the present exploratory study with small sample size we observed only a moderate impact of long-term quinolone prophylaxis on the intestinal and sputum microbiome with no relevant long-term volatility after the initiation of antibiotic prophylaxis. However, we report remarkable inter-individual differences of the microbiome between patients, and high frequencies of *Enterobacteriaceae* (> 20%) during antibiotic prophylaxis might indicate failure of or non-adherence to quinolone prophylaxis. This pilot study is the first to implicate that gut microbiome monitoring may be useful to detect prophylaxis failure in patients with liver advanced liver cirrhosis and could be a helpful tool to tailor individual prophylactic strategies in these patients. However, our results may not be clinically significant given the limitations of the study and therefore future studies are urgently needed to further investigate this phenomenon.

**Table 1.** Patients’ characteristics

Characteristics	Patients (n = 11)
Age, years, mean (range)	64 (57–72)
Male sex, n (%)	7 (63.6)
Etiology of cirrhosis	
Alcohol, n (%)	9 (81.8)
NASH, n (%)	1 (9.1)
Other, n (%)	1 (9.1)
Severity and complication of cirrhosis	
MELD-score	20 (± 9)
Child Pugh B, n (%)	3 (27.3)
Child Pugh C, n (%)	8 (72.7)
Esophageal varices, n (%)	5 (45.5)
Prior gastrointestinal bleeding, n (%)	3 (27.3)
Prior hepatic encephalopathy n (%)	1 (9.1)
Prior hepatorenal syndrome, n (%)	1 (9.1)
Primary SBP prophylaxis, n (%)	6 (54.5)
Fluoroquinolones used for prophylaxis	
Norfloxacin, n (%)	8 (72.7)
Ciprofloxacin, n (%)	3 (27.3)
Concomitant medication	
Beta-blocker, n (%)	6 (54.5)
Proton-pump inhibitors, n (%)	7 (63.6)
Laboratory results	
C-reactive protein (mg/dL)	2.9 (± 2.7)
White blood count (/nL)	7.8 (± 3.0)
Bilirubin (mg/dL)	5.9 (± 6.5)
Alanine aminotransferase (U/L)	40 (± 37)
International normalized ratio	1.8 (± 0.5)
Creatinine (mg/dL)	1.5 (± 0.9)
Albumin (g/dL)	3.2 (± 1.5)

## Supplementary information

**Additional file 1: Figure S1.** Principal coordinates analysis (PCoA) of weighted and unweighted UniFrac distances (A, B) as well as bacterial richness and Shannon’s index diversity with respect to different time points (C, D) and inter-individual differences (E, F) of stool RNA.

## Data Availability

Date on which the conclusions of the manuscript rely are available within the manuscript and Additional file. Raw dataset of stool analysis are available upon request as rma-files (due to the large file size).
