# Supplementary material for: Stool and sputum microbiome during quinolone prophylaxis of spontaneous bacterial peritonitis: an exploratory study
Source: Gut Pathog. 2020 Oct 30;12:51. doi: 10.1186/s13099-020-00389-y (PMC7596951; doi:10.1186/s13099-020-00389-y)
Supplement: Supplementary file 1 — Additional file 1: Figure S1. Principal coordinates analysis (PCoA) of weighted and unweighted UniFrac distances (A, B) as well as bacterial richness and Shannon’s index diversity with respect to different time points (C, D) and inter-individual differences (E, F) of stool RNA. [file 13099_2020_389_MOESM1_ESM.docx]

**Additional File 1**

**Stool and sputum microbiome during quinolone prophylaxis of spontaneous bacterial peritonitis**

Marcus M. Mücke^1^, Sabrina Rüschenbaum^1,2^, Amelie Mayer^1^, Victoria T. Mücke^1^, Katharina M. Schwarzkopf^1^, ^,^ Stefan Zeuzem^1^, Jan Kehrmann^3,#^, René Scholtysik^4,#^, Christian M. Lange^1,2,#^

**Affiliations:**

^1^Department of Internal Medicine 1, University Hospital Frankfurt, Frankfurt am Main, Germany

^2^Department for Gastroenterology and Hepatology, University Hospital Essen and University of Duisburg-Essen, Essen, Germany (current address)

^3^Institute of Medical Microbiology, University Hospital Essen, University of Duisburg-Essen, Essen, Germany

^4^Institute of Cell Biology, University Hospital Essen and University of Duisburg-Essen, Essen, Germany

^#^These authors have contributed equally to this manuscript.

**Figure S1**


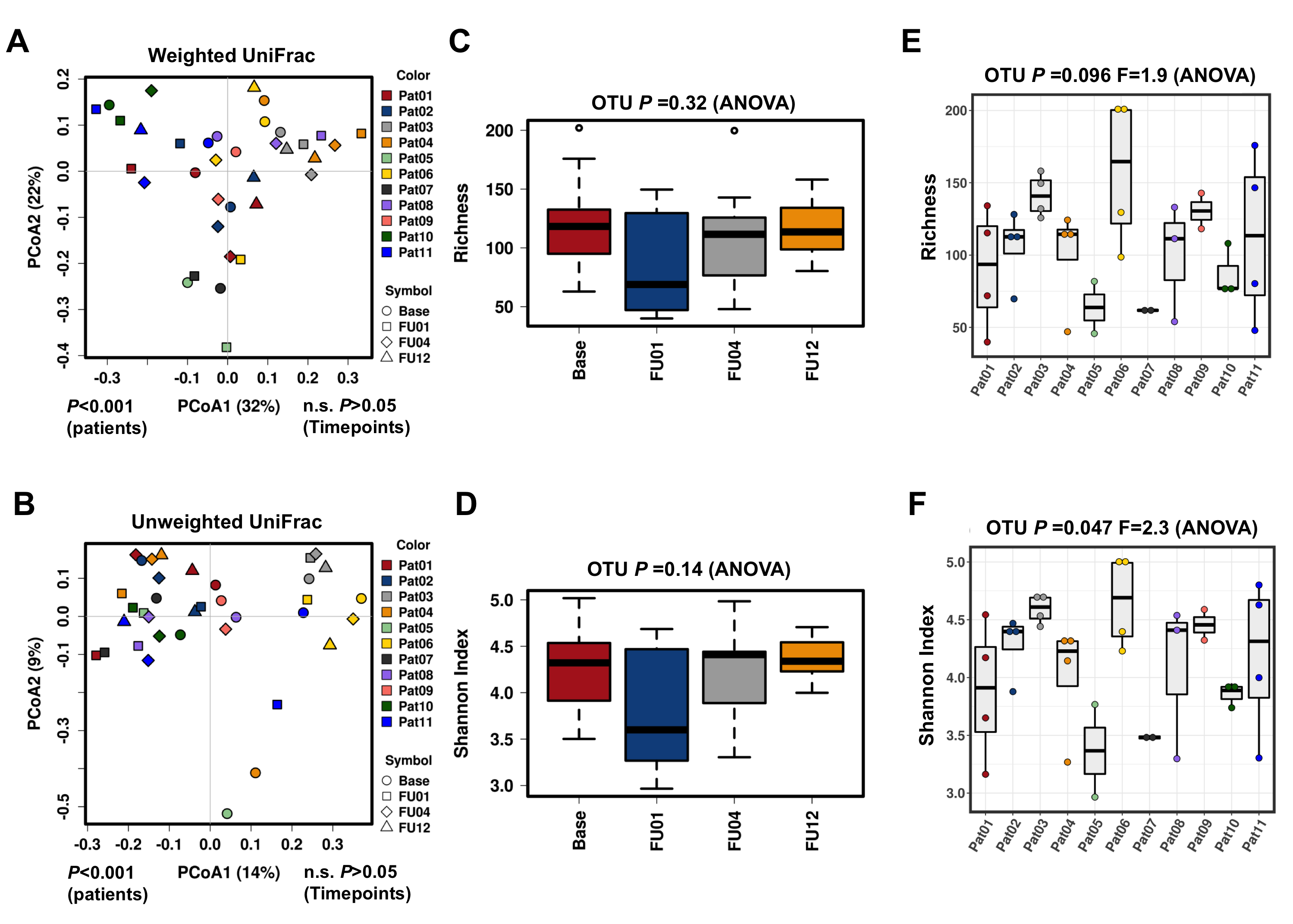


**Figure S1.** Principal coordinates analysis (PCoA) of weighted and unweighted UniFrac distances (A, B) as well as bacterial richness and Shannon‘s index diversity with respect to different time points (C,D) and inter-individual differences (E,F) of stool RNA.
